# An international study of factors affecting variability of dosimetry calculations, part 5: impact of segmentation methods

**DOI:** 10.1186/s40658-026-00848-6

**Published:** 2026-03-12

**Authors:** Sara Kurkowska, Yuni K. Dewaraja, Eric C. Frey, Julia Brosch-Lenz, John Sunderland, Carlos Uribe

**Affiliations:** 1 Department of Nuclear Medicine, Pomeranian Medical University, Szczecin, Poland; 2 Department of Integrative Oncology, BC Cancer Research Institute, Vancouver, BC Canada; 3 Department of Radiology, University of Michigan, Ann Arbor, MI USA; 4 Rapid, LLC, Baltimore, MD USA; 5 Department of Radiology, Johns Hopkins University, Baltimore, MD USA; 6 Institute of Nuclear Medicine, Glen Burnie, MD USA; 7 Department of Radiology, University of Iowa, Iowa City, IA USA; 8 Department of Molecular Imaging and Therapy, BC Cancer, Vancouver, BC Canada; 9 Department of Radiology, University of British Columbia, Vancouver, BC Canada

**Keywords:** ^177^Lu, Dosimetry, Segmentation, Radiopharmaceutical therapies, SPECT/CT

## Abstract

**Background:**

Individualized radiopharmaceutical therapies guided by patient-specific absorbed dose assessments using imaging have the potential to improve both efficacy and safety. Understanding sources of variability in absorbed dose calculations is critical for standardization. The Society of Nuclear Medicine and Molecular Imaging Dosimetry Task Force launched the ^177^Lu Dosimetry Challenge to evaluate variability across different steps within the dosimetry workflow. This work aimed to assess the variability in absorbed doses due to differences in segmentation methods.

**Methods:**

Anonymized datasets from two patients treated with ^177^Lu-DOTATATE, including serial SPECT/CT scans, were made available online. Participants were asked to segment healthy organs and lesions and perform dosimetry calculations. In a subsequent task, participants were provided with standardized segmented VOIs and asked to perform dosimetry based on these pre-defined regions. Variability in segmentation was assessed by comparing absorbed dose estimates across two scenarios: participant-generated segmentations versus predefined reference segmentations. Relative absorbed dose variability was quantified using the quartile coefficient of dispersion (QCD) and interquartile range.

**Results:**

Variability in absorbed dose (measured as QCD difference between absorbed doses from participant-generated segmentations and those from reference segmentations) for kidneys was less than 5% in simple cases and 10.6% for more challenging scenarios (i.e. presence of intraparenchymal cysts, cortical defects). Lesion segmentation exhibited higher variability, with absorbed dose variability reaching up to 22.4%.

**Conclusions:**

Segmentation significantly contributes to variability in absorbed dose estimates, particularly for lesions and for kidneys with anatomical complexities. Standardizing segmentation protocols and providing training on advanced segmentation methods are essential to reduce variability.

**Supplementary Information:**

The online version contains supplementary material available at 10.1186/s40658-026-00848-6.

## Background

Radiopharmaceutical therapies (RPTs), particularly ^177^Lu-DOTATATE and ^177^Lu-PSMA, have seen increasing use in recent years and could potentially be used earlier in the treatment course. While initially approved for patients with disease progressing after standard systemic therapies [1, 2], recent FDA approval of ^177^Lu-PSMA for use after androgen receptor pathway inhibitor therapy, but prior to texane-based chemotherapy, based on the PSMAfore trial [3], reflects this shift. Beyond this, trials such us PSMAddition (NCT04720157) are evaluating ^177^Lu-PSMA in patients with metastatic hormone-sensitive prostate cancer, signaling continued expansion of RPT into earlier disease settings [4].

A key characteristic of ^177^Lu-labeled radiopharmaceuticals is their emission of beta radiation for therapy and gamma-rays for imaging with Single Photon Emission Computed Tomography (SPECT), enabling both visualization and absorbed dose estimation. However, absorbed dose estimation from SPECT imaging involves a complex, multi-step process, with variability in methods contributing to inconsistent dose estimates. These inconsistencies hinder meaningful comparisons of dosimetry results across clinical centers and development of generalizable absorbed dose outcome models. Addressing this variability is critical to advancing RPT dosimetry.

To identify sources of variability, the Society of Nuclear Medicine and Molecular Imaging (SNMMI) Dosimetry Task Force initiated the Lutetium-177 (^177^Lu) Dosimetry Challenge. This initiative aimed to identify and quantify the variability across the dosimetry workflow through five tasks, each designed to isolate a specific step of the workflow. The first three tasks assessed how different imaging protocols contributed to absorbed dose variability by comparing SPECT/CT, planar, and hybrid approaches [5]. Two additional tasks provided participants with VOIs and TIA maps to eliminate variability related to segmentation and time integration, allowing assessment of variability in segmentation, time-activity curve fitting [6] and absorbed dose calculation [7]. This work evaluates how segmentation methods affect absorbed dose calculations, offering data-driven recommendations to enhance consistency in clinical practice.

## Methods

### Data collection

Two patients treated with ^177^Lu-DOTATATE (7.21 GBq for patient A, 7.31 GBq for patient B) underwent four post-therapeutic SPECT/CT scans to track radiopharmaceutical distribution and kinetics at 3.7 h, 27.7 h, 103.1 h and 124.0 h post-injection for patient A and 3.7 h, 32.6 h, 99.6 h and 193.3 h for patient B. All SPECT/CT data were acquired on a Siemens Symbia Intevo system using three energy windows (186–227 keV main, 165–186 keV and 227–248 keV scatter). Each acquisition included 120 projections per window. Images were reconstructed with xSPECT Quant (48 iterations, 1 subset, no post-reconstruction filter) with attenuation, scatter, and collimator–detector response corrections applied. Further details are provided in [8]. Pre-therapeutic diagnostic images, along with quantitative SPECT images and metadata are accessible via the University of Michigan's Deep Blue repository (https://deepblue.lib.umich.edu/data) [9, 10]. Patient A’s diagnostic images included an abdominal magnetic resonance (MR) scan and a ^68^Ga-DOTATOC PET/CT performed 9 and 15 months before therapy, respectively. Patient B’s images included contrast-enhanced abdominal and pelvic CT (venous and arterial phases) and a ^68^Ga-DOTATOC PET/CT performed 3.5 months and 2 months before therapy, respectively.

Participants used the provided quantitative SPECT images to calculate absorbed dose for kidneys, healthy liver, spleen (except patient B due to splenectomy) and specified lesions (two for patient A, four for patient B). Relevant intermediary metrics and calculations were reported using preformatted spreadsheets, which detailed volume of interest (VOI) segmentation methods and the subsequent dosimetry workflow. Axial, sagittal, and coronal images displaying organ and lesion segmentations were also submitted. Participants were instructed that there was no need to apply partial volume corrections (PVCs) but to describe PVC procedures if used.

### Data analysis

To evaluate the impact of segmentation on absorbed dose calculations, we conducted a three-step analysis. Step 1 categorized the segmentation methods used by participants. Step 2 quantified segmentation-induced variability by comparing absorbed dose variability when participants performed segmentation (task 1) versus when segmentation was provided (task 4). Finally, Step 3 consisted of analyses performed by the authors to investigate potential sources of the variability observed in participants results in Step 2. Below, we describe each step in more detail.

#### Step 1: Analysis of segmentation methodology

We categorized and recorded the frequency of segmentation methods used, classifying them as:

1. Manual: defined entirely by an operator.
2. Threshold: based on a fixed threshold value (e.g. percentage of maximum voxel value) applied to SPECT, CT, or absorbed dose map.
3. Threshold + manual: threshold-based segmentation refined manually.
4. Sphere: small spherical VOI placed within the organ.
5. Semi-automatic: combined manual and automated processes (e.g. deformable registration algorithms) to assist across slices.
6. Gradient: algorithms based on intensity gradients.
7. Automatic, artificial intelligence (AI)-based: deep-learning models trained on labeled data for VOI segmentation.
8. Automatic, atlas-based: applied contours from pre-contoured CT atlases after registration.

We recorded whether diagnostic or only post-therapeutic SPECT/CT images were used for segmentation, noting the specific imaging dataset (SPECT, CT component of SPECT/CT, or dose map) used. Time estimates for segmentation and methods for mass estimation were also noted.

For kidney segmentations, we documented whether participants excluded renal pelvis and cysts (present in both kidneys of patient A and the right kidney of patient B). This was evaluated by reviewing orthogonal images included in the submitted spreadsheets. While the provided screenshots often did not capture all cysts in full, they were sufficient to assess the general approach to segmentation. Extraparenchymal cysts, typically easier to identify on CT, served as a reliable indicator for overall approach to cyst exclusion. Based on this, we applied the following classification:

1. Including all cysts—if the VOI clearly contained extraparenchymal cysts, suggesting no attempt was made to exclude any cysts;
2. Partially including cysts—if extraparenchymal cysts were excluded, but at least one intraparenchymal cyst (which is more subtle and harder to detect, especially on low-resolution CT) remained included;
3. Excluding cysts—if even the intraparenchymal cysts were excluded, indicating exclusion of all visible cysts.

Ambiguous methodologies were flagged, and segmentation methods were grouped separately for healthy organs and lesions, reflecting consistent methodology for the segmentation of each group.

#### Step 2: Quantifying segmentation-induced variability in absorbed dose estimates provided by participants

We quantified variability using two methods. The first method analyzed data from all participants grouped by task, facilitating comparisons with other Dosimetry Challenge articles. The second method used patient-specific paired analyses, which accounted for and canceled out covariance effects, enabling more robust comparisons of segmentation techniques when analyzed collectively. All variability metrics were calculated using the complete dataset without exclusion of any values. Quartile-based measures were chosen because the distributions were non-normal and these statistics are robust to skew and outliers.

Relative variability in absorbed dose due to segmentation approaches was evaluated by comparing results from tasks 1 and 4. In task 1, participants independently performed segmentation. For task 4, organizers provided predefined VOIs in both RTStruct and binary mask formats. Organ masks were segmented on CT at each time point using the Contour ProtégéAI+ model, a U-Net-based convolutional neural network within MIM Software (performance of this model is available in [11]). These AI-generated VOIs excluded the pelvis and 3 (out of 6) cysts but incorrectly included intraparenchymal cysts, such as one in the left kidney of patient A and two in right kidney of patient B. Lesion masks were manually segmented by a radiologist on the first time point CT and transferred to subsequent time points. The dosimetry workflow steps were identical for both tasks 1 and 4.

We analyzed data from participants who completed both tasks, assessing absorbed dose variability using the quartile coefficient of dispersion (QCD), calculated as the difference between the 75th and 25th percentiles divided by their sum. The difference in QCD between tasks 1 and 4 quantified variability when no segmentation was required. VOIs were provided in two formats, (RTStruct and binary masks) to enhance accessibility, but differences in activity and volume between these formats inadvertently introduced additional variability in task 4 data. To address this, variability in task 4 was assessed separately for each VOI format, and a weighted QCD (QCDw) was calculated as:

1 $$ {\mathrm{QCD}}_{{\mathrm{w}}} = \frac{{n_{RTStruct} \cdot QCD_{RTStruct} + n_{mask} \cdot QCD_{mask} }}{{n_{total} }}, $$

where n_RTStruct_ is the number of submissions using VOIs provided in RTStruct format, n_mask_ is the number of submissions using VOIs provided in binary mask format, and n_total_ is the total number of submissions (n_RTStruct_ + n_mask_). QCD_RTStruct_ refers to the QCD for the RTStruct subset and QCD_mask_ refers to the QCD for the mask subset.

The difference in QCDw between tasks 1 and 4 was referred to as the overall variability.

Additionally, we performed a paired analysis to examine participant-specific and method-specific variability. For each participant, the segmentation method used in task 1 was identified, and we calculated:

$$ \Delta {\mathrm{AD}}/\overline{{{\mathrm{AD}}}}_{{{\mathrm{task}}4}} = \frac{{{\mathrm{AD}}_{{{\mathrm{task}}1}} - {\text{ AD}}_{{{\mathrm{task}}4}} }}{{\overline{{{\mathrm{AD}}}}_{{{\mathrm{task}}4}} { }}} \cdot 100\% , $$

where ${\mathrm{AD}}_{{{\mathrm{task}}1}}$ and ${\mathrm{AD}}_{{{\mathrm{task}}4}}$ are the absorbed doses obtained by each participant in tasks 1 and 4, respectively, $\overline{{{\mathrm{AD}}}}_{{{\mathrm{task}}4}}$ is the mean absorbed dose of task 4 ($\overline{{{\mathrm{AD}}}}_{{{\mathrm{task}}4}}$) across participants. ΔAD reflected the absorbed dose difference between participant-defined VOIs and those provided in task 4. $\Delta {\mathrm{AD}}/\overline{{{\mathrm{AD}}}}_{{{\mathrm{task}}\;4}}$ serves as a measure of variability introduced by the segmentation techniques. Positive values indicate higher absorbed doses in task 1, and values may exceed ± 100% when participant-defined segmentations produce absorbed doses more than double (or less than half) of those from the standardized VOIs. The interquartile range (IQR) of $\Delta {\mathrm{AD}}/\overline{{{\mathrm{AD}}}}_{{{\mathrm{task}}4}}$ distribution was used to assess variability, and a weighted IQR (IQRw) was calculated analogously to Eq. 1. These normalized values were grouped and analyzed by segmentation method category.

#### Step 3: Quantifying segmentation-induced variability in absorbed dose estimates generated by the authors

The findings from previous steps identified sources of variability due to segmentation methods. In this step, we conducted a controlled analysis to investigate how different segmentation methods introduce variability. Using the RTStruct-format VOIs provided in task 4 as a reference, all additional segmentations (described below) were performed by a physician (SK) using MIM software v7.2.1 (MIM Software, GE Healthcare). The absorbed dose calculations differed only by segmentation method; subsequent dosimetry calculations used the same biokinetic model, integration and dosimetry methods. The Scipy Python library [12] was used for nonlinear least squares fitting with the Levenberg–Marquardt algorithm and MIRDcalc v1.1 [13] to calculate absorbed dose values from time integrated activity coefficients (TIACs).

##### Kidneys

In participants’ submissions, we observed inconsistent handling of the renal pelvis and cysts. To assess how inclusion or exclusion of these structures affects absorbed dose estimates, we first refined the task 4 AI-generated VOIs under physician (SK) supervision to ensure that all cysts were excluded. This new set was defined as the refined reference. From this reference, two modified VOIs were generated:

1. The refined reference VOI with the renal pelvis added, and
2. The refined reference VOI with all cysts added.

For each VOI configuration, we determined recovered activity (with no PVC performed), the volume (mean of four time points), TIAC, absorbed dose, and percentage differences to determine relative to the refined reference. Absorbed dose variations were also analyzed using volumes from individual scans instead of the mean. The same VOI was used to calculate both activity and mass, with no PVC applied to maintain comparability with participant results.

Similarly, the challenge instructions requested an estimate of absorbed dose to healthy liver tissue alone. However, some participants included intra-hepatic lesions in their liver VOIs, leading to overestimated absorbed doses, as discussed in previous dosimetry challenge articles [9, 10]. The spleen was not investigated in Step 3 due to little variability observed in Step 2.

##### Lesions

Threshold-based segmentation using SPECT data was the most common approach for lesion delineation, with participants applying a wide range of percentage thresholds. To evaluate the impact of threshold values on absorbed dose estimates for lesions, we applied thresholds ranging from 10 to 75% in 5% increments. We compared two strategies:

1. Independent thresholding, applying thresholds individually to each time point,
2. Threshold propagation, applying a threshold on the second SPECT image, then propagating the resulting VOI to subsequent time points with positional adjustments but no reshaping.

We also assessed two methods for estimating lesion masses, both assuming a uniform density of 1.0 g/mL:

1. Constant mass approach using a fixed mass derived from the CT-based VOI provided in task 4, independent of SPECT threshold variations. Here, we recreate participants that apply threshold on SPECT image to estimate activity (the segmented SPECT VOI varied with the applied threshold), but the mass is taken from CT (therefore CT-based mass remained constant and does not change with the threshold).
2. Variable mass approach, calculating mass directly from the SPECT VOI volume, which changed with the applied threshold. In this case, we recreate participants that using a threshold generate a single VOI, from which both activity and mass were calculated.

## Results

### Step 1: Analysis of segmentation methodology

**Fig. 1:**
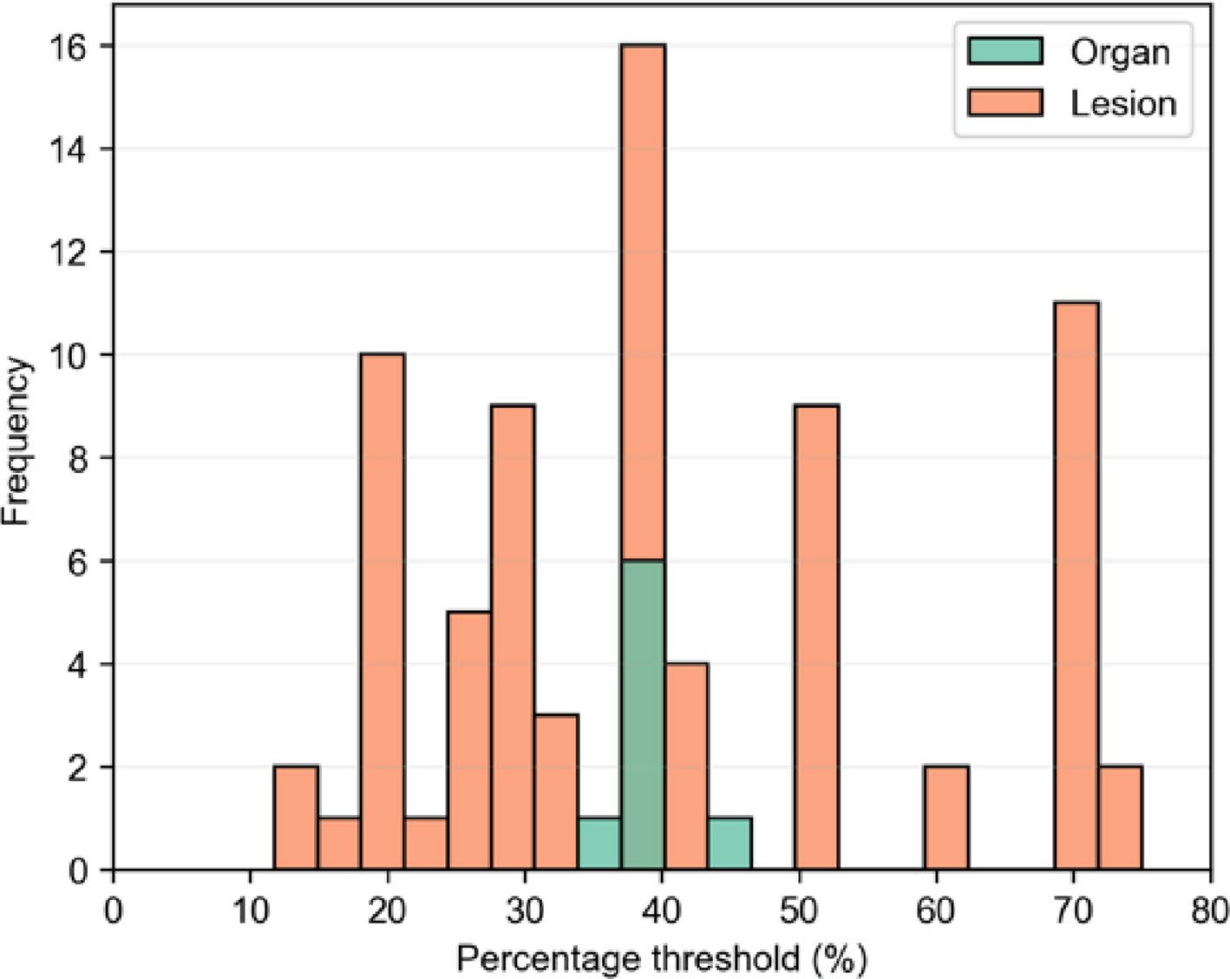
Histogram of thresholds values applied to SPECT images

In task 1, different segmentation methods were used across 52 independent groups (102 total submissions, with 51 for patient A and 51 for patient B). This analysis focuses on segmentation methods, with the trends summarized in Table 1, which provides the total number of segmentations, percentages, and median segmentation times for organs and lesions per method. Results are aggregated across both patients and all organs and lesions to show the overall trends. Manual segmentation was the most common approach for healthy organs and threshold-based methods for lesions. Among threshold methods, 82% used the SPECT image, 17% the CT, and 1% the dose map. Thresholds (on SPECT) ranged from 11 to 75% for lesions and 35% to 45% for organs, as shown in Fig. 1. Segmentation time varied, with manual methods being the most time-consuming.

**Table 1:** Summary of segmentation methods, the frequency (percentage and number of cases) and time required for each segmentation method applied to healthy organs and lesions

Segmentation method	Healthy organs		Lesions	
	Frequency (% and n)	Time in min (median (range))	Frequency (% and n)	Time in min (median (range))
Manual	53.5% (170)	28.2 (2.0–299.0)	25.3% (67)	26.2 (1.0–90.0)
Threshold	2.8% (9)	11.7 (5.0–20.0)	33.2% (88)	15.4 (2.0–60.0)
Threshold + Manual	15.1% (48)	19.7 (5.0–53.0)	15.5% (41)	15.4 (3.0–30.0)
Sphere Method	11.6% (37)	5.9 (1.0–30.0)	9.1% (24)	1.7 (1.0–3.0)
Semi-automatic	11.3% (36)	18.8 (1.8–45.0)	2.3% (6)	2.0 (2.0–2.0)
Gradient	–	–	14.7% (39)	0.7 (0.1–2.0)
Automatic: AI-based	4.7% (15)	4.0 (2.0–8.0)	–	–
Automatic: atlas-based*	0.9% (3)	10.0 (10.0–10.0)	–	–

*Applied only for liver segmentation

**Fig. 2:**
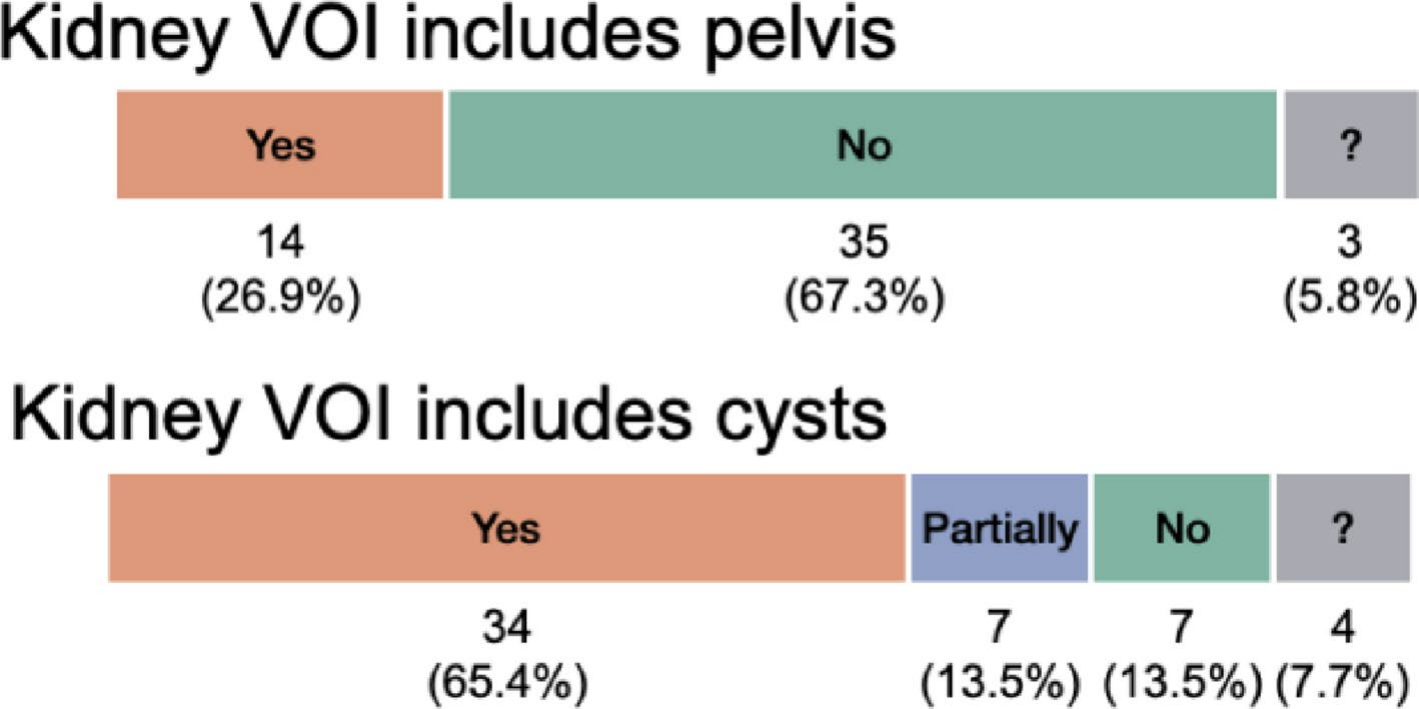
The number and percentage of participants in task 1 who included the renal pelvis and cysts within the kidney VOIs

Figure 2 shows the percentage of submitters who included the renal pelvis or cysts within the kidney VOIs. Most (67.3%) excluded the renal pelvis, segmenting only the renal cortex and medulla. However, 65.4% included clearly visible extraparenchymal cysts, indicating a tendency to include all cysts, while 13.5% included some but not all.

Supplemental Tables 1 to 3 provide additional data about the primary image type used for segmentation, and methods for estimating mass, volume, and density.

### Step 2: quantifying segmentation-induced variability in absorbed dose estimates provided by participants

**Fig. 3:**
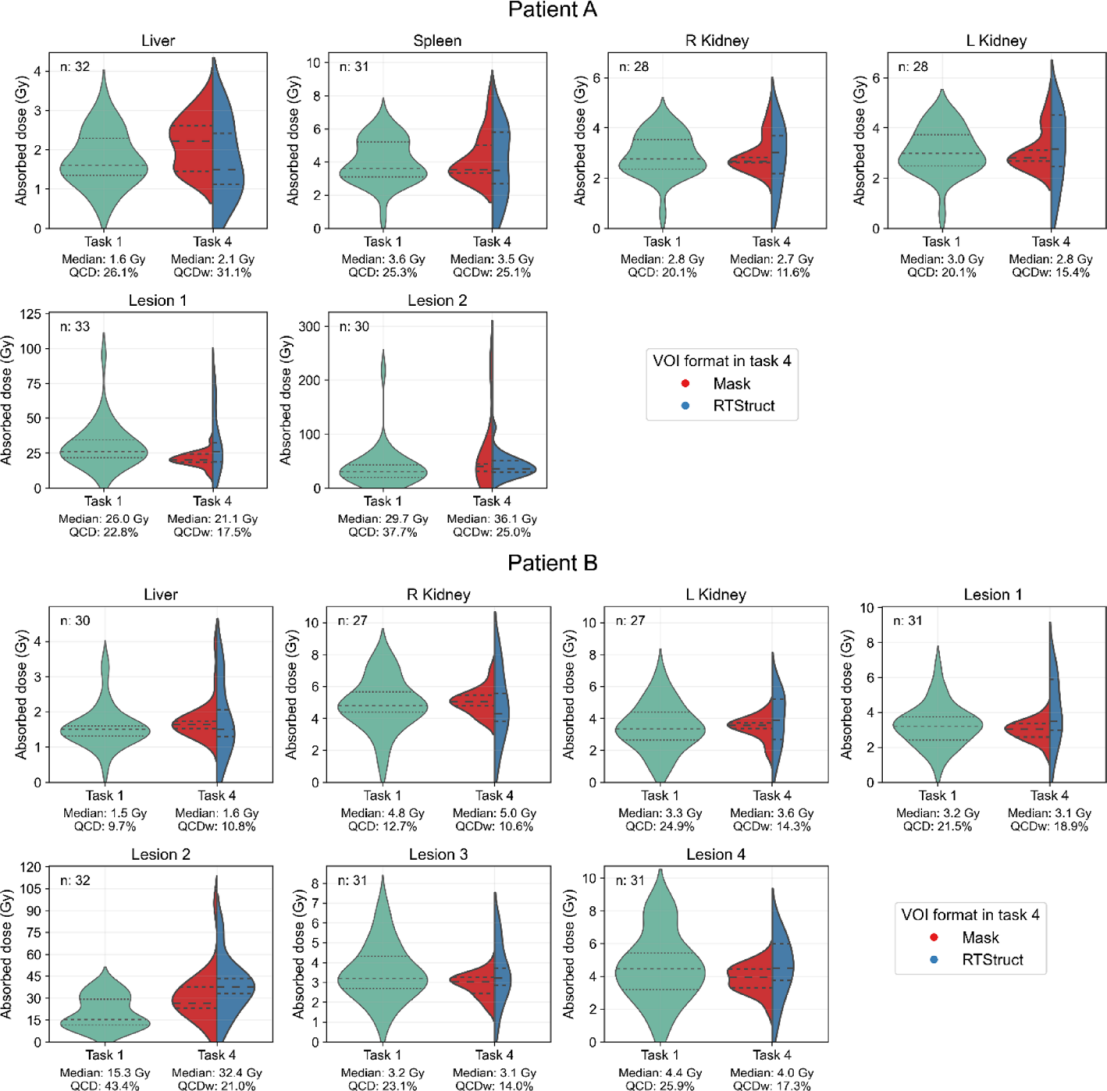
Distribution of absorbed doses per region reported by participants with submissions for both tasks 1 and 4; median, QCD, and number of submissions per organ (n) are indicated in each boxplot

We analyzed 65 submissions from participants who completed both tasks 1 and 4 (33 for patient A and 32 for patient B). Figure 3 shows violin plots of absorbed doses for each region, with horizontal lines indicating quartiles and the median. Below each plot, the median and QCD are noted. For task 4, datasets from mask and RTStruct VOI formats are also included. Table 2 compares the QCD of absorbed dose from task 1 and the QCDw from task 4, showing how absorbed dose variability (QCD) changed when segmentation was provided. The QCD was higher in task 1 for kidneys and lesions, but comparable for the spleen, suggesting minimal segmentation-induced variability. For the liver, higher variability in task 4 arose from inconsistent handling of tumors, with some participants including them in liver volume segmentation while others excluded them. The variability was small for patient B (13.5 mL of intra-hepatic lesions) but larger for patient A (110.7 mL), highlighting the impact of these discrepancies.

**Table 2:** Difference in QCD of absorbed dose (%) between task 1 and 4 for each region, evaluated using quartiles of the distributions presented in Fig. 3

	Difference in QCD (%)^*^	
Region	Patient A	Patient B
Liver	− 5	− 0.8
Spleen	0.2	–
R Kidney	8.5	2.1
L Kidney	4.7	10.6
Lesion 1	5.3	3.0
Lesion 2	11.3	22.4
Lesion 3	–	10.4
Lesion 4	–	8.7

*QCD task 1 minus QCDw task 4

**Fig. 4:**
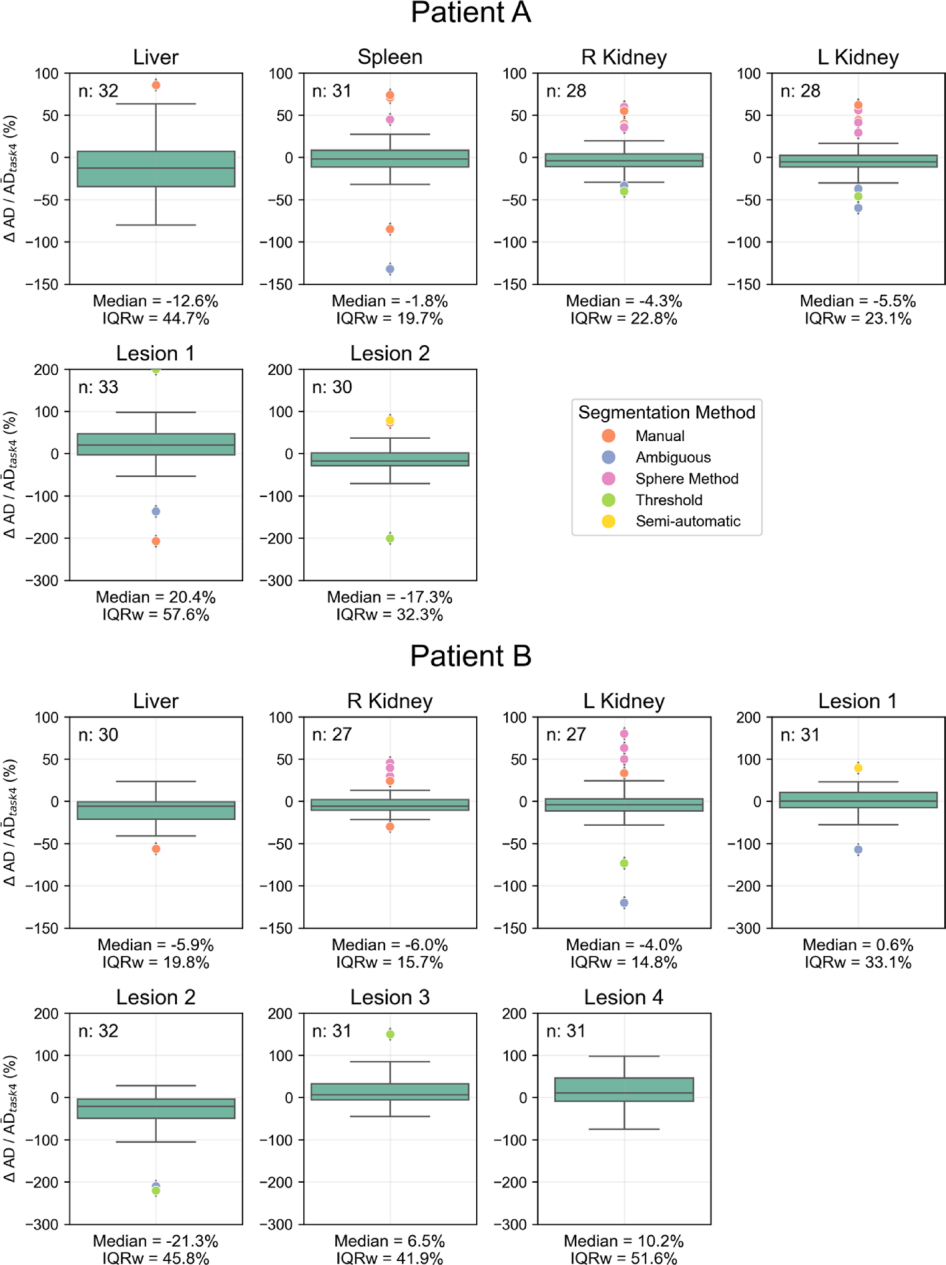
Distribution of ΔAD/$\overline{{{\mathrm{AD}}}}_{{{\mathrm{task}}4}}$ values calculated for all regions using absorbed doses reported by participants with submissions for both tasks; median, IQR, and number of submissions (n) are indicated in each boxplot. Outliers are color-coded to differentiate between different segmentation methods

**Fig. 5:**
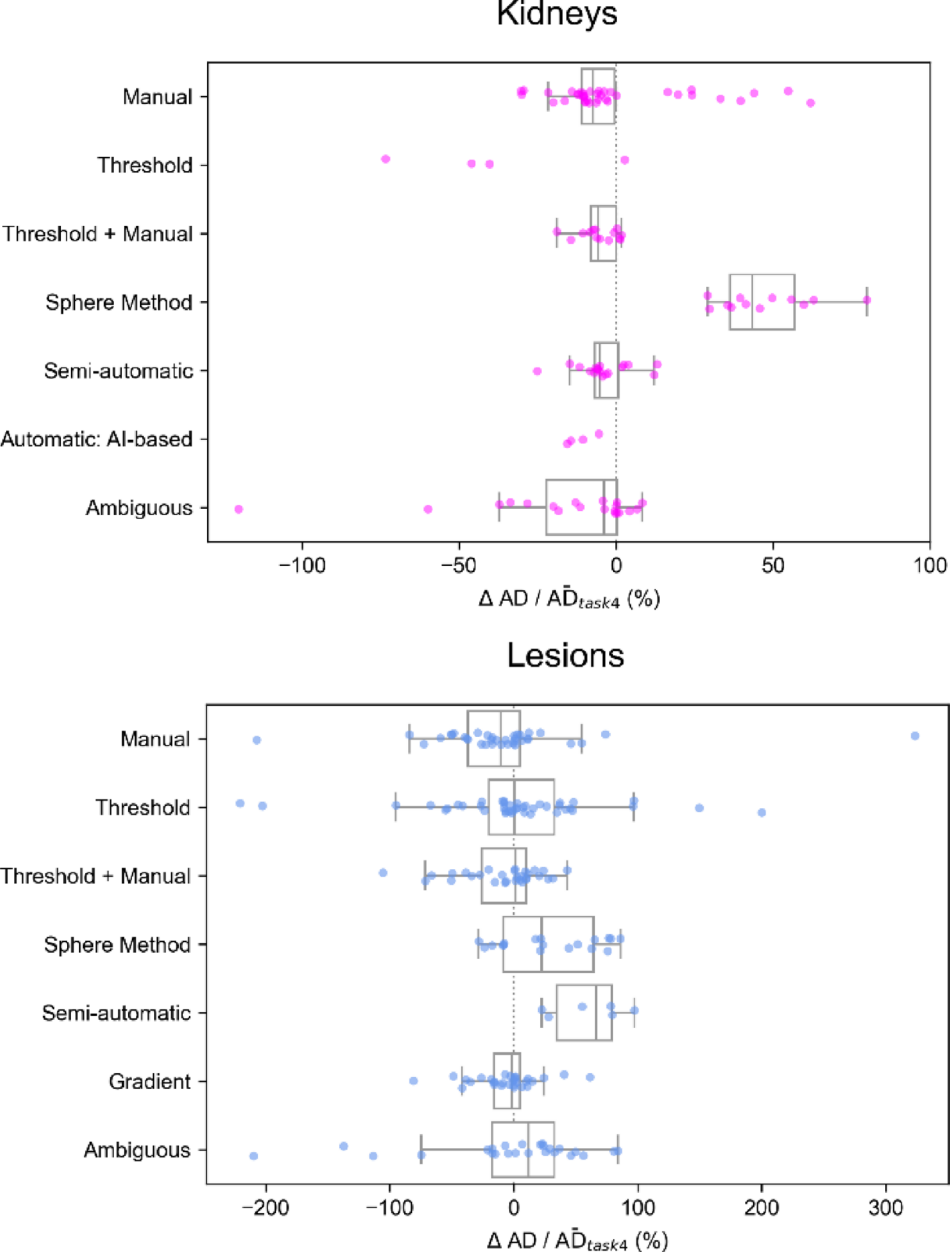
Comparison of segmentation methods for kidneys and lesions based on ΔAD/${\overline{\mathrm{AD}}}_{\mathrm{task}4}$. The top panel shows the results for different segmentation methods applied to kidneys, and the bottom panel presents the results for lesions

Figure 4 presents $\Delta {\mathrm{AD}}/\overline{{{\mathrm{AD}}}}_{{{\mathrm{task}}\;4}}$ with median and IQR, color-coded for outliers by segmentation method. Most outliers came from the same submissions. Figure 5 and Table 3 show the performance of various segmentation methods for kidneys and lesions. For kidneys manual, threshold + manual, semi-automatic, and AI-based methods had mean values within 15% of the reference. AI-based method showed the lowest variability and threshold and sphere methods exhibited the greatest differences and variability (however, both AI-based and threshold-based methods were not commonly used for kidneys, which limits the generalizability of the findings for these approaches). For lesions, gradient methods had the lowest IQRw, while the sphere method had the highest, with all methods showing relatively high IQRw values. The semi-automatic method had the highest median difference (with only 6 examples being studied). Results for liver and spleen are presented in Supplemental Fig. 1 and 2. The analysis on the impact of including/excluding intra-hepatic lesions from the liver can be found in the previous paper [7].

**Table 3:** Median $\Delta {\mathrm{AD}}/\overline{{{\mathrm{AD}}}}_{{{\mathrm{task}}4}}$ (%), IQR (%) and number (n) for each segmentation method for kidneys and lesions

Segmentation method	Kidneys				Lesions			
	Median	IQR	Range	n	Median	IQR	Range	n
Manual	− 7.4	10.6	92.1	50	− 10.3	42.1	530.7	38
Threshold	− 43.2	23.4	76.2	4	0.4	52.8	420.7	46
Threshold + Manual	− 5.7	8.1	20.7	18	1.0	35.7	148.5	30
Sphere Method	43.5	20.3	50.8	16	22.6	72.7	114.4	18
Semi-automatic	− 5.2	7.7	38.3	18	66.5	44.4	74.5	6
Gradient	–	–	–	–	− 1.7	21.0	142.1	30
Automatic: AI-based	− 12.5	5.3	9.9	4	–	–	–	–
Ambiguous	− 4.0	22.4	128.5	20	11.6	49.9	294.1	25

### Step 3: quantifying segmentation-induced variability in absorbed dose estimates generated by the authors

#### Kidney

Table 4 provides sample cross-sectional images illustrating the following VOIs datasets: task 4 as provided, refined reference standard (task 4 VOIs adjusted by a physician), refined reference standard with renal pelvis included, refined reference standard with renal cysts included. Table 5 summarizes the mean volumes, TIACs, and absorbed doses for each scenario, along with percentage differences demonstrating the impact of including cysts and the renal pelvis on these metrics. The variability in absorbed dose estimates resulting from the use of different VOI volumes is depicted in Supplemental Fig. 3.

**Table 4:**
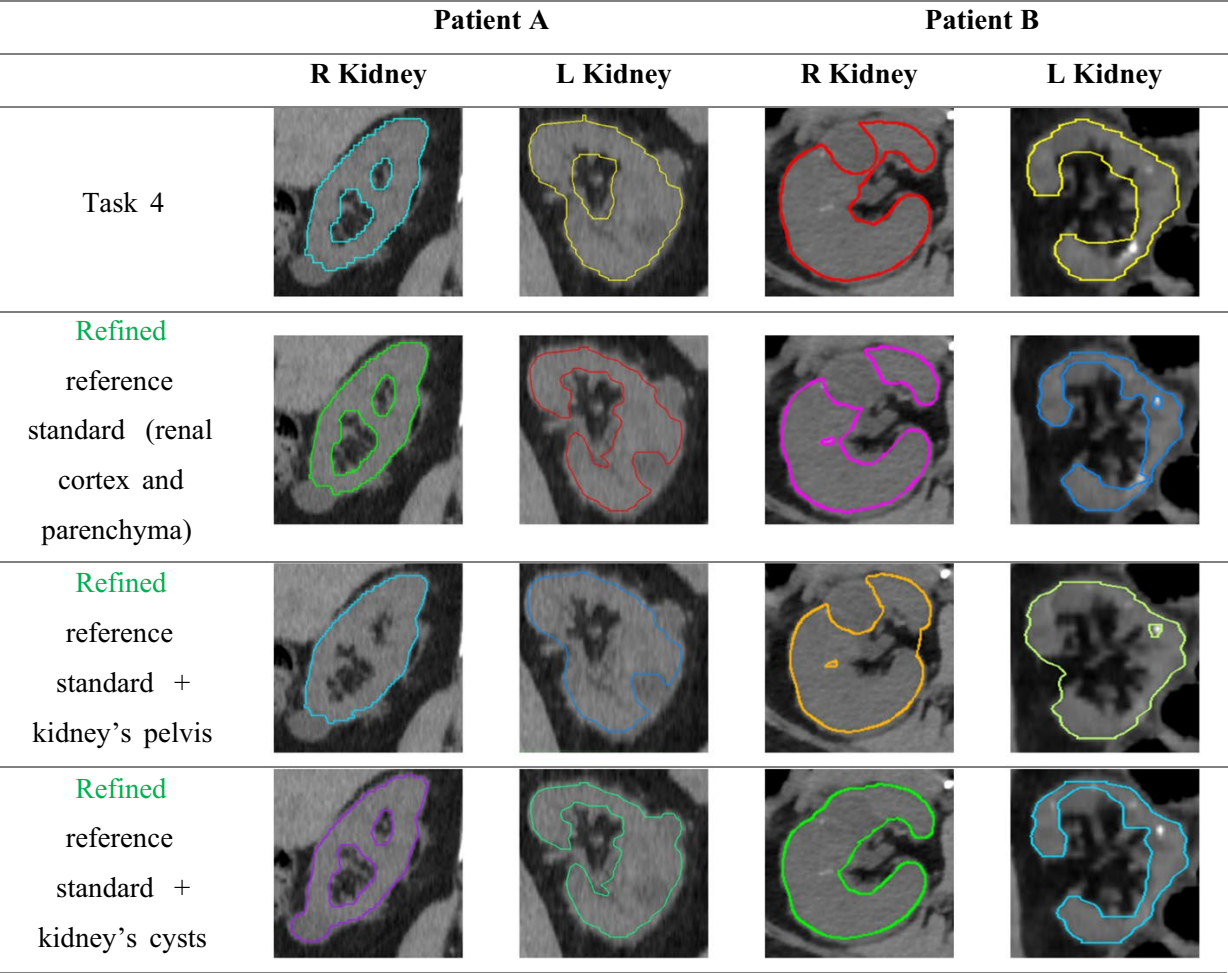
Examples of VOIs for the indicated segmentation scenarios

**Table 5:** Mean volumes, TIACs, absorbed dose evaluated for kidneys from the different VOI scenarios*

		Patient A		Patient B	
		R Kidney	L Kidney	R Kidney	L Kidney
Refined reference standard (renal cortex and parenchyma)	Mean volume (mL)	192.9	195.9	151.32	66.38
	TIAC (h)	0.85	0.94	1.29	0.43
	AD (Gy)	2.75	3.01	5.37	4.02
Refined reference standard + kidney’s pelvis	Mean volume (mL)	225.6 (17.0%)	236.9 (21.0%)	186.8 (23.4%)	97.55 (46.9%)
	TIAC (h)	0.91 (7.1%)	1.00 (5.9%)	1.48 (14.5%)	0.54 (27.3%)
	AD (Gy)	2.52 (− 8.4%)	2.64 (− 12.2%)	4.99 (− 7.1%)	3.49 (− 13.2%)
Refined reference standard + kidney’s cysts	Mean volume (mL)	205.3 (6.4%)	203.7 (4.0%)	163.57 (8.1%)	67.46 (1.6%)
	TIAC (h)	0.86 (0.7%)	0.97 (1.8%)	1.33 (2.7%)	0.43 (0.23%)
	AD (Gy)	2.60 (− 5.5%)	2.94 (− 2.2%)	5.11 (− 4.9%)	3.98 (− 1.3%)

AD, absorbed dose

*Percentage differences indicate the difference in the metric from the reference case for the given scenario

#### Lesions

Table 6 summarizes the characteristics of lesions included in the challenge, detailing the following: (a) diagnostic imaging modalities (MR for patient A and contrast-enhanced CT for patient B), (b) CT component of SPECT/CT at time point 1 used by the radiologist to delineate lesion VOIs, (c) SPECT/CT at time points 1 and 2, (d) SUVmax values from the second time point quantitative SPECT, (e) lesion volumes, (f) tumor-to-background ratios (TBR, calculated as lesion SUVmax divided by the SUVmean of healthy liver tissue).

**Table 6:**
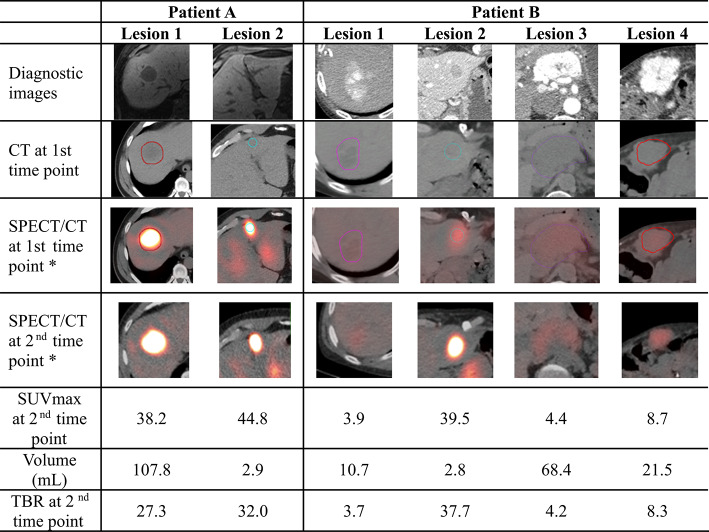
Lesion characteristics in the challenge dataset

*SUV threshold upper limit 20

The SUVmax ranged from 3.9 to 39.5, lesion volumes from 2.8 to 108 mL, and TBR from 3.7 to 37.7, showcasing significant variability in lesion characteristics within the dataset.

**Fig. 6:**
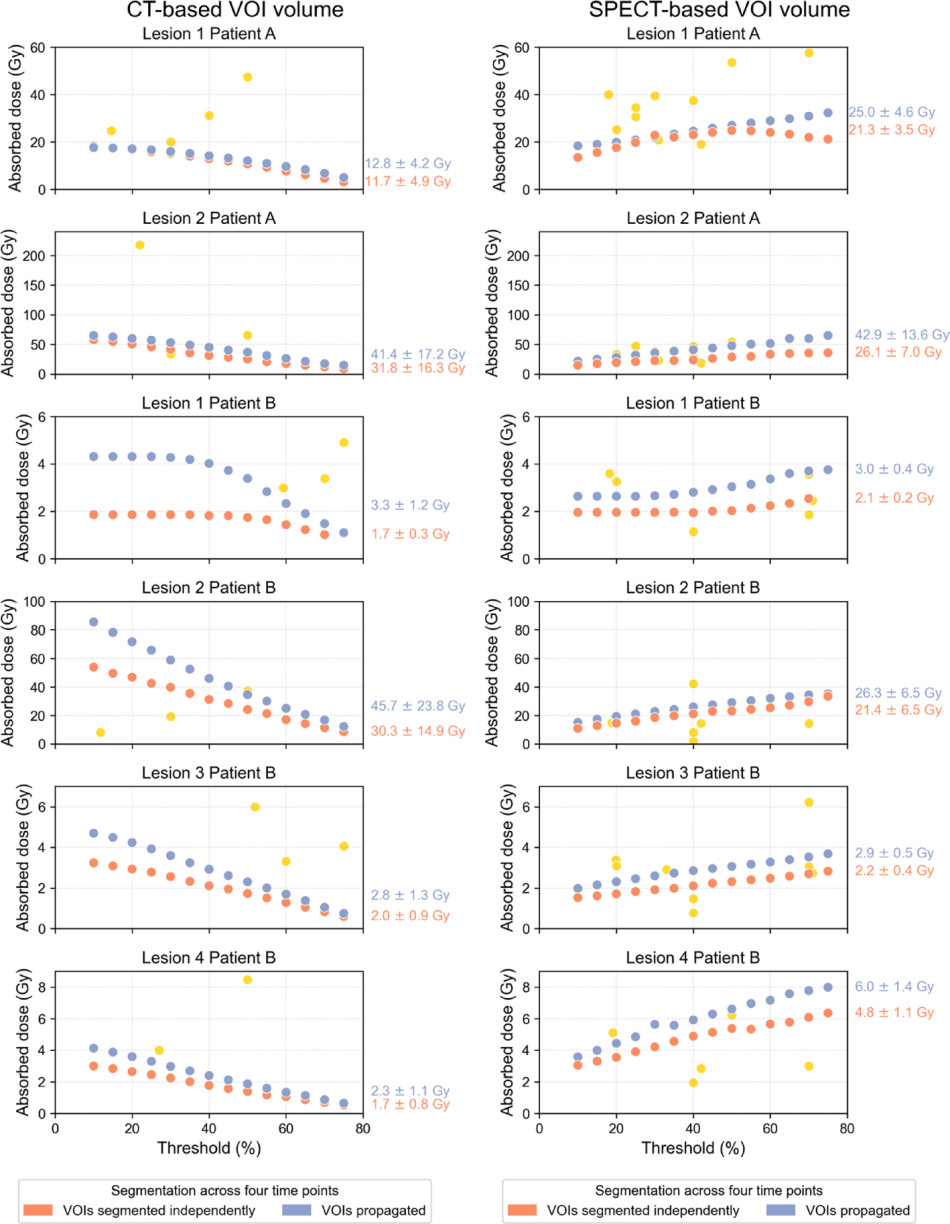
Results of absorbed dose estimates generated by the authors showing the impact of different thresholding methods. The left column represents participants who applied thresholds on SPECT images to estimate activity, while the mass was taken from CT and remained constant (independent of SPECT threshold changes). The right column represents participants who used thresholds to create a single VOI from which both activity and mass were calculated, resulting in different estimated masses for different thresholds. Blue dots represent the threshold applied to the second SPECT scan and propagated to subsequent time points; orange dots represent thresholds applied independently to each SPECT scan; and yellow dots indicate participant-reported absorbed dose from those using the threshold-based methods (categorized based on whether they reported using CT- or SPECT-based VOI volumes). The means and standard deviations are shown for each method.

Figure 6 shows the effect of varying thresholds on absorbed dose estimates in a controlled analysis. Two scenarios were evaluated: (1) lesion mass derived from CT-based VOIs (left column) and (2) lesion mass derived from SPECT-based VOIs (right column). Blue dots represent thresholds applied to the second SPECT scan and propagated to other time points, orange dots are thresholds applied independently to each SPECT scan, and yellow dots participant-reported values using threshold-based methods. We observe high variability in absorbed dose when recreating the range of threshold used by participants. Regarding absorbed dose trends, for CT-based VOIs, absorbed dose estimates decreased with increasing thresholds and for SPECT-based VOIs increased. In Lesion 1 of Patient A of SPECT-based VOIs, the absorbed dose decreases at higher thresholds because, at low to moderate thresholds, lesion mass decreases faster than activity, whereas above this threshold, total activity declines more rapidly than mass, leading to a reduction in absorbed dose. Absorbed doses were generally higher for propagated VOIs than for independently segmented VOIs. Participant reported absorbed dose estimates (yellow dots) occasionally fell outside the range calculated by the authors, likely due to differences in the CT-estimated volumes, curve fitting, integration methods, and absorbed dose conversion. Variability introduced by these steps is reported in [6, 7]. Supplemental Fig. 4 shows the impact of thresholds on VOI volumes. CT-based volumes defined by the radiologist serve as reference, and no single threshold consistently produced volume estimates matching those of the radiologist across all lesions.

## Discussion

This study investigated the variability in absorbed dose estimates in RPTs caused by different segmentation methods. Unlike prior studies, which typically assess inter-operator variability within single institutions or compare a limited set of segmentation techniques in isolation [14–18], our approach incorporates over 102 submissions from 52 independent centers. These submissions, applied to a common dataset of two patients reflect a wide range of real-world segmentation practices. For example, Ilan et al. reported lower variability under highly standardized phantom conditions [17], whereas the present challenge analyzed patient data across multiple groups using their routine segmentation workflows. This design intentionally captures the heterogeneity of current clinical practice, explaining the higher observed variability and highlights the need for future harmonization efforts. This international scale is essential, as studies confined to single institutions or countries often underestimate variability due to higher intra-institutional consistency and shared local practices. We note that some extreme values observed in this analysis may reflect isolated participant errors rather than intrinsic method variability. Therefore, we do not consider range a generalizable measure of variability, but we fully acknowledge that in therapeutic dosimetry, outliers could potentially have clinical consequences. Some participants were relatively unexperienced in dosimetry, and reporting individual misapplications would not add generalizable scientific value. Our focus is therefore on trends and consistently misapplied practices that affect multiple participants.

The task 4 VOIs were not intended to represent a ground-truth reference, but rather to provide a standardized comparator that reduces segmentation variability on the results for that task. Differences between participant segmentations and task 4 VOIs reflect methodological differences, such as the use of small VOIs versus whole-organ VOIs, rather than true errors. This design allowed us to isolate the effect of methodological choices on absorbed dose estimates.

Discussion of some findings in this paper is limited by the lack of information on whether participants would normally apply PVC. For example, some participants segmented organs on CT to estimate mass and then expanded the VOI to estimate activity, while others used a single VOI for both mass and activity. The latter is acceptable only if followed by PVC. We also acknowledge that different segmentation methods may vary in their dependence on PVE and PVC, which we could not evaluate in this study; for instance, a sphere-based method is less influenced by PVE. However, the data from this challenge revealed a practice that, in the authors' view, is suboptimal: approximately 12% of submissions reported activity VOI volumes that were smaller than the corresponding mass VOI volumes (Supplemental Table 2). This suggests the use of a high threshold to segment only the highest-activity regions, which likely leads to an underestimation of the absorbed dose. While one could argue this could be followed by PVC, we advocate for a methodology where a consistent segmentation—generally aiming for an activity VOI that is larger than or equal to the anatomical VOI—is established first. A validated PVC method should then be applied explicitly to this well-defined volume. This sequential approach improves reproducibility and avoids the potential for errors. Additionally, different PVC methods, discussed, for example, in the work of Tran-Gia and Lassman [19] could introduce additional variability, but this source of variability was not evaluated in this work.

The time required for segmentation is a significant practical consideration in the dosimetry workflow. Reported data (Table 1) confirm that it can be highly time-consuming, particularly for manual methods (median: 28.2 min; with one liver segmentation requiring 299 min). This confirms that segmentation is time-intensive and a major contributor to the overall time. It also shows that automated tools can substantially reduce the effort required.

Spleen segmentation showed minimal variability and is therefore not discussed here, although results are detailed in the supplementary material. Liver segmentation posed challenges due to difficulties in isolating segmentation effects because the user decision to include or exclude liver lesions in liver volume overwhelmed segmentation impact, which was analyzed in previous paper [7]. The focus of this discussion is kidneys and lesions, as reducing variability in these regions is crucial for improving our understanding of dose-toxicity and dose–response relationships for this RPT. Kidney absorbed dose variability due to segmentation was influenced by anatomical challenges including cysts and cortical defects. In cases with minimal anatomical challenges, the overall variability due to segmentation (as measured by the difference in QCD for tasks 1 and 4) in absorbed dose was less than 5% (Table 2). However, in more complex scenarios, this variability increased to 10.6% (Table 2). Lesion segmentation exhibited higher variability, with absorbed dose variability reaching up to 22.4% (Table 2). Factors contributing to the observed variability should inform strategies to minimize segmentation-related variability.

### Kidney variability

Overall absorbed dose variability due to different segmentation methods (measured by QCD difference) was highest in the left kidney of patient B and the right kidney of patient A (Table 1), likely due to cortical defects and cyst contributions, respectively. Participants inconsistently included or excluded the renal pelvis and/or cysts, which significantly impacted variability. In this dataset, including the renal pelvis increased the VOI volume by up to 46.9% and TIAC by 27.3%, reducing the absorbed dose by 13.2%. At the initial imaging time point, SPECT images showed activity in the renal pelvis from urinary excretion, contributing to TIAC. However, including the renal pelvis in the VOI disproportionately increases the organ mass, which can lead to underestimation of absorbed dose to the functional renal tissue. According to EANM recommendations, SPECT image segmentation and VOIs should preferably encompass the kidney regions that retain activity over time, i.e. the cortex and medulla [20]. Including cysts, which exhibit no radiopharmaceutical uptake or minimal spill-in from surrounding kidney tissue, similarly inflated organ mass and underestimated absorbed dose up to 5.1%. These findings underscore the need for consistent VOI delineation, focusing on functional renal parenchyma, following guidelines to reduce variability and improve accuracy.

The sphere method resulted in systematically higher absorbed dose estimates, with a median ΔAD/$\overline{{{\mathrm{AD}}}}_{{{\mathrm{task}}4}}$ of 43.5%. This discrepancy may reflect differences in how VOIs were defined across tasks—for example, the use of small VOIs (without PVE issue) in task 1 compared with whole-kidney VOIs without PVC in task 4. Hou et al. [21], found that placing a sphere in the kidney’s high-activity region overestimated absorbed dose by a mean factor of 1.8 compared with whole-kidney segmentation followed by PVC via expansion. Their hybrid method combined SPECT-derived activity with CT-derived volume, potentially including non-functional areas such as cysts. These findings emphasize the need for consistent anatomical and functional VOI definitions when relating activity concentration to absorbed dose. Sandström et al. [18] reported good agreement among the sphere, threshold, and anatomical methods in a single-center study using a calibrated protocol. In contrast, the present multi-center challenge allowed participants to use unharmonized methods, revealing substantial variability. The sphere method high variability with IQRw of 20.3% (Table 3) observed in this work highlights the critical importance of standardized procedures to achieve the reproducibility demonstrated by Sandström et al.

### Lesion variability

Segmentation-induced variability in absorbed dose in lesions reached up to 22.4%, with the highest variability observed for lesion 2 of patient B, which although it had the highest TBR one could think it would make the segmentation more consistent, this lesion was also the smallest. Threshold-based segmentation, the most commonly used method, exhibited significant variability (52.8%, Table 3), second only to the sphere method. This variability stemmed from the wide range of thresholds used (11% to 75%), particularly in lesions with high SUVmax and TBR. Although a fixed threshold of 42% is the most commonly used approach for SPECT-based segmentation, our findings, consistent with those of Gustafsson et al. [22], indicate that this method does not always provide an accurate estimation of lesion activity or volume (Supplemental Fig. 4), particularly in heterogeneous lesions with regions of low uptake.

Absorbed dose estimates replicated by the authors showed notable sensitivity to threshold choice. For CT-based VOIs, higher thresholds reduced TIAC and absorbed dose, while for SPECT-based VOIs, increasing thresholds simultaneously decreased mass and activity but increased absorbed dose due to a higher activity concentration in the lesion center. The fact that some participants' data fell outside the estimated range is due to other steps in the workflow that can also introduce significant variability.

The optimal thresholds depend on lesion size, TBR, and imaging resolution among other factors. Prior studies [23] suggest ~ 30% thresholds for larger volumes with ^177^Lu, but fixed thresholds for patient tumors are prone to inaccuracies due to heterogeneous radionuclide uptake and variable background activity. Furthermore, for small objects, the limited spatial resolution of SPECT/CT prevents the activity concentration from reaching the true maximum value within the voxels. System-specific phantom measurements are recommended for optimizing thresholds based on object size. CT-based mass assessment combined with PVC methods appears to be a promising approach.

Lesion segmentation ideally requires diagnostic quality CT or MR evaluation, often demanding physician expertise. However, only three institutions in this study involved physicians in the dosimetry workflow. Most participants did not use diagnostic images for segmentation (only 26.9% used it when segmenting organs and 29.9% when segmenting lesions), despite their value in refining anatomical delineation. While the CT component of SPECT/CT provides anatomical information, low-dose CT protocols, lack of contrast, and larger voxel sizes hinder fine feature identification. For example, higher resolution CT scan at time point 1 allowed better visualization of renal cysts compared to subsequent low-dose scans. Similarly, AI-based kidney segmentation on lower-resolution CT scans resulted in VOIs up to 20% smaller, primarily missing portions near the liver or cysts (Supplemental Fig. 5).

Characterization of absorbed dose variability due to segmentation was influenced by several limitations in this study. First, the small sample size, limited to two patients, reduces the generalizability of the findings. However, the dataset captured common challenges encountered in clinical practice, such as difficulties in delineating lesions on CT, variability in volumes and uptakes, and the presence of anatomical complexities like cysts and cortical defects. The performance and variability of segmentation methods depend on reconstruction and imaging protocols. In this work, we did not evaluate the variability introduced by different reconstruction methods or settings; instead all participants were provided with a single set of reconstructed images to ensure comparability across institutions. Nonetheless, different reconstruction choices may increase or reduce the variability of some segmentation methods compared with what was observed here.

Additionally, since the study used patient data with unknown true absorbed dose values, the focus was solely on variability rather than accuracy. Finally, while task 4 aimed to minimize segmentation-related variability, discrepancies were observed between VOIs derived from RTstructs and binary masks, suggesting potential differences in how these formats are handled within the software to estimate mass and activity.

Based on the findings of this study, we propose the following data-driven segmentation-related actions to minimize variability and ensure consistency in absorbed dose estimates:

1. Standardize the definition of functional renal regions: Consistency in identifying functional renal parenchyma (cortex and medulla) is critical. Structures such as the renal pelvis, cysts, or lesions within the organ should be excluded. Our analysis demonstrated that including the renal pelvis or cysts led to absorbed dose values up to 13.2% lower than the refined reference.
2. The functional (activity) VOI used should fully encompass the anatomical (mass) VOI, in both organs and tumors, to ensure that all activity contributing to the absorbed dose is included. Defining an activity VOI smaller than the corresponding mass VOI risks excluding peripheral activity regions and underestimating absorbed dose. Consistent alignment between anatomical and functional VOIs improves reproducibility and minimizes systematic bias in dose estimation.
3. Reevaluate the use of the sphere method: Given its high variability (20.3% assessed for kidneys by IQR) the sphere method requires further optimization and harmonization before routine use. Without standardization, this method may introduce unnecessary variability especially when the activity in the anatomical region is not uniform.
4. Leverage high-resolution imaging for anatomical VOIs: Whenever available, high-resolution imaging, such as diagnostic CT or MRI acquired in close temporal proximity to therapeutic scans, should be used to define anatomical VOIs for mass estimation. This can include a higher-dose CT acquired as part of post-therapeutic SPECT/CT. These imaging modalities offer greater anatomical detail compared to low-dose or contrast-limited scans. For example, our analysis showed that applying the same AI-based kidney segmentation method to lower-resolution CT scans reduced organ volumes by up to 20% and failed to exclude cysts or other features reliably. Importantly, functional imaging remains the most critical tool for identifying tissue that does or does not express the therapeutic target.
5. Threshold-based segmentation remains highly variable, highlighting the need for continued collaborative efforts to establish common approaches for threshold selection and validation. Building on the alignment principles in recommendation #2, future studies should aim to define harmonized procedures that ensure accurate and reproducible activity and mass estimation.

## Conclusions

This study highlights the critical role of segmentation in the dosimetry workflow, as demonstrated through the SNMMI ^177^Lu Dosimetry Challenge data. Kidney absorbed dose variability was closely linked to segmentation complexity and anatomical factors, the presence of such as cysts or cortical defects. While variability remained below 5% in straightforward cases, it increased to 10.6% in more challenging scenarios, underscoring the need for standardized procedures, particularly for patients with baseline impairments. Lesion segmentation exhibited even greater variability, up to 22.4%, emphasizing the importance of harmonized practices to ensure reproducible dosimetry. Implementing the recommendations outlined in this work can significantly reduce variability and enhance the reliability of absorbed dose estimates. Standardized segmentation practices are pivotal not only for improving precision but also for advancing the personalization of radiopharmaceutical therapies, ultimate optimizing patient outcomes.

## Supplementary Information

Below is the link to the electronic supplementary material.

Supplementary Material 1

## Data Availability

The dataset analyzed during the current study are available from corresponding author on reasonable request.

## Abbreviations

AI: Artificial intelligence
CT: Computed tomography
IQR: Interquartile range
IQRw: Weighted IQR
MR: Magnetic resonance
PVC: Partial volume correction
QCD: The quartile coefficient of dispersion
QCDw: Weighted QCD
RPT: Radiopharmaceutical therapy
SNMMI: Society of nuclear medicine and molecular imaging
SPECT: Single photon emission tomography
SUV: Standardized uptake value
TBR: Tumor-to-background ratio
TIA: Time integrated activity
TIAC: TIA coefficient
VOI: Volume of interest
