# Supplementary material for: An international study of factors affecting variability of dosimetry calculations, part 5: impact of segmentation methods
Source: EJNMMI Phys. 2026 Mar 12;13:39. doi: 10.1186/s40658-026-00848-6 (PMC13096261; doi:10.1186/s40658-026-00848-6)
Supplement: Supplementary file 1 — Supplementary Material 1 [file 40658_2026_848_MOESM1_ESM.docx]

**An International Study of Factors Affecting Variability of Dosimetry Calculations, Part 5: Impact of Segmentation Methods**

**GRAPHICAL ABSTRACT**


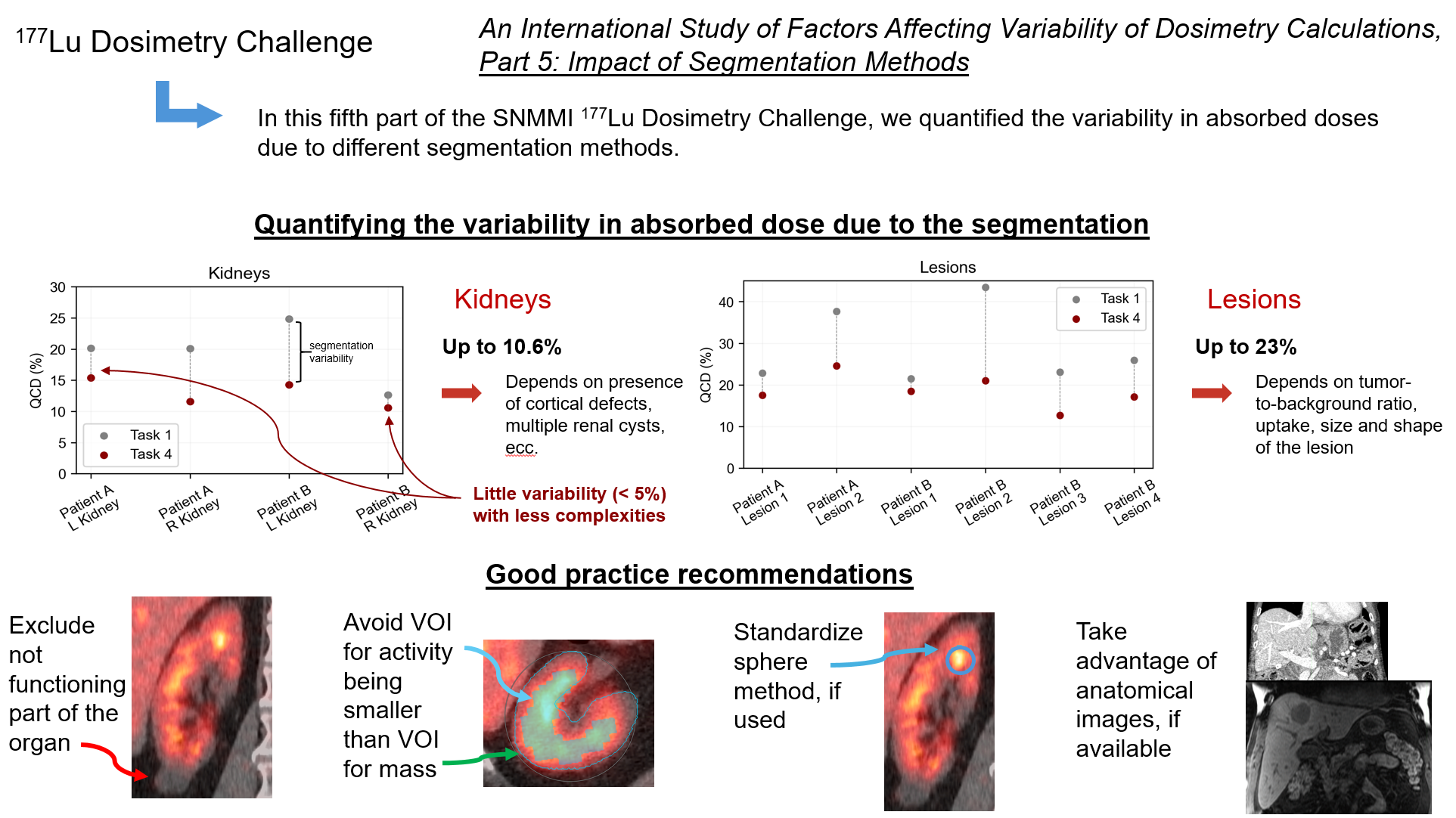


**SUPPLEMENTARY MATERIALS**

Supplemental Table 1 presents data on the use of the provided diagnostic images as guidance tools for segmenting healthy organs and lesions, as well as the primary imaging modality used for segmentation. Overall, diagnostic images were not frequently used for segmentation guidance. The CT component of SPECT/CT was predominantly used as the primary image for healthy organ segmentation, whereas SPECT was more commonly used for lesion segmentation.

**Supplemental Table 1:** Percentage (%) and number of healthy organs and lesions segmented with the assistance of diagnostic images (PET/CT or MR) and the primary imaging modality used for segmentation.

|  | **Healthy organs** | **Lesions** |
| --- | --- | --- |
| **Usage of diagnostic images as a guidance during segmentation** | | |
| Yes | 26.9% (88) | 29.9% (83) |
| No | 73.1% (239) | 70.1% (195) |
| **Image that was primarily used for segmentation** | | |
| SPECT | 19.1% | 79.9% |
| CT | 80.9% | 17.5% |
| Dose map | 0% | 2.6% |

Supplemental Table 2 shows different methods used to estimate mass, volume and density. Only a few participants used mass values derived from the literature, and these participants were the ones employing the sphere method. In most cases (approximately 70%), participants used a single Volume of Interest (VOI) to obtain both activity and mass. This was the case for both lesions and normal organs. Others used separate VOIs for activity and mass determination. Roughly half of participants assumed the density to be constant (the most common one was 1 g/mL, followed by 1.05 g/mL and 1.03 g/mL) and other half measured it from CT (for healthy organs densities ranged from 1.0 to 1.12 g/mL and lesions 1.0 to 1.07 g/mL). Detailed information on densities used are shown in Supplemental Table 3.

**Supplemental Table 2**: Different method used to calculate mass for healthy organs and lesions.

| **Category** | **Subcategory** | **Healthy Organs** | **Lesions** |
| --- | --- | --- | --- |
| **Mass** | mass derived from literature | 16 | 6 |
|  | mass calculated using formula:  mass = volume x density | 324 | 286 |
| **Volume** | VOI activity > VOI mass | 28 | 29 |
|  | VOI activity = VOI mass | 213 | 181 |
|  | VOI activity < VOI mass | 37 | 34 |
|  | 4 ml sphere | 37 | 24 |
| **Density** | constant density | 127 | 118 |
|  | density derived from CT scans | 116 | 100 |

**Supplemental Table 3:** Summary of different densities used to assess the mass

|  |  | Number | |
| --- | --- | --- | --- |
|  | Density (g/mL) | Healthy organs | Lesions |
| Constant density | 1 | 79 | 70 |
|  | 1.03 | 7 | 18 |
|  | 1.045 | - | 6 |
|  | 1.05 | 27 | 24 |
|  | 1.052 | 4 | - |
|  | 1.06 | 3 | - |
|  | 1.066 | 4 | - |
|  | 1.079 | 2 | - |
|  | 1.089 | 1 | - |
| CT based | 1.0-1.12 | 116 | - |
|  | 1.0-1.07 | - | 100 |


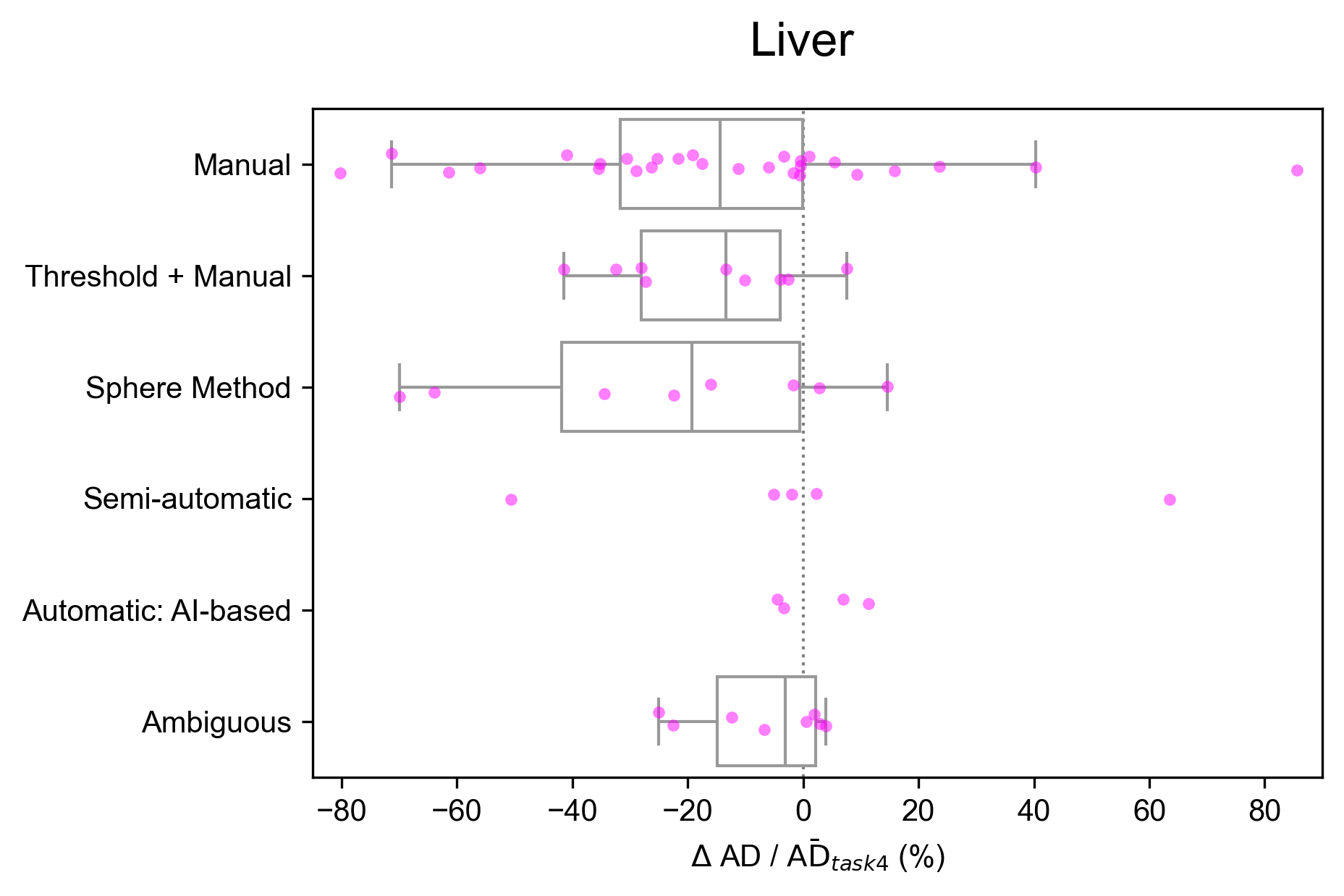


**Supplemental Figure 1**: Comparison of segmentation methods for liver based on ΔAD/${\overline{\mathrm{AD}}}_{task4}$.

**Supplemental Figure 2**: Comparison of segmentation methods for spleen based on ΔAD/${\overline{\mathrm{AD}}}_{task4}$.


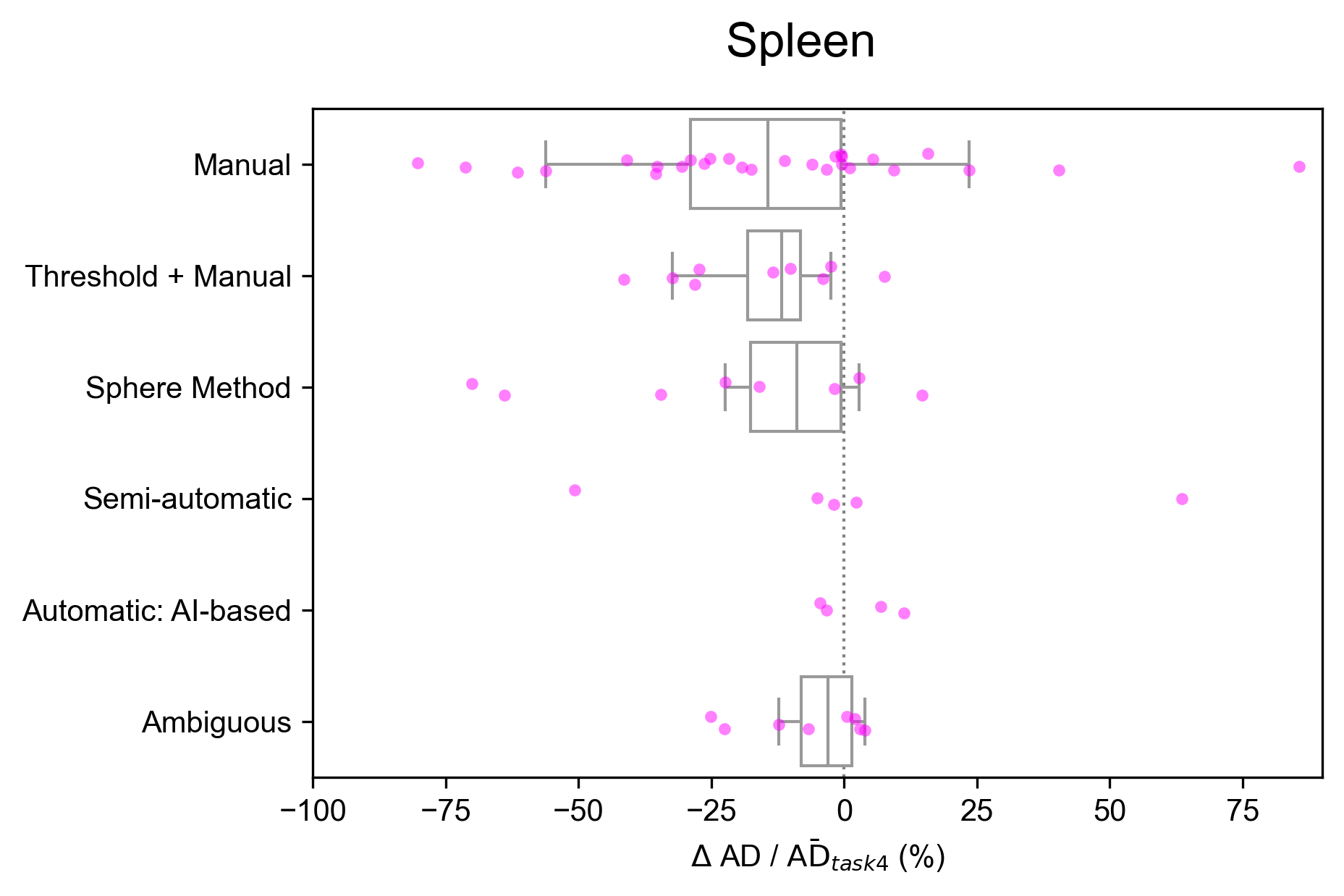


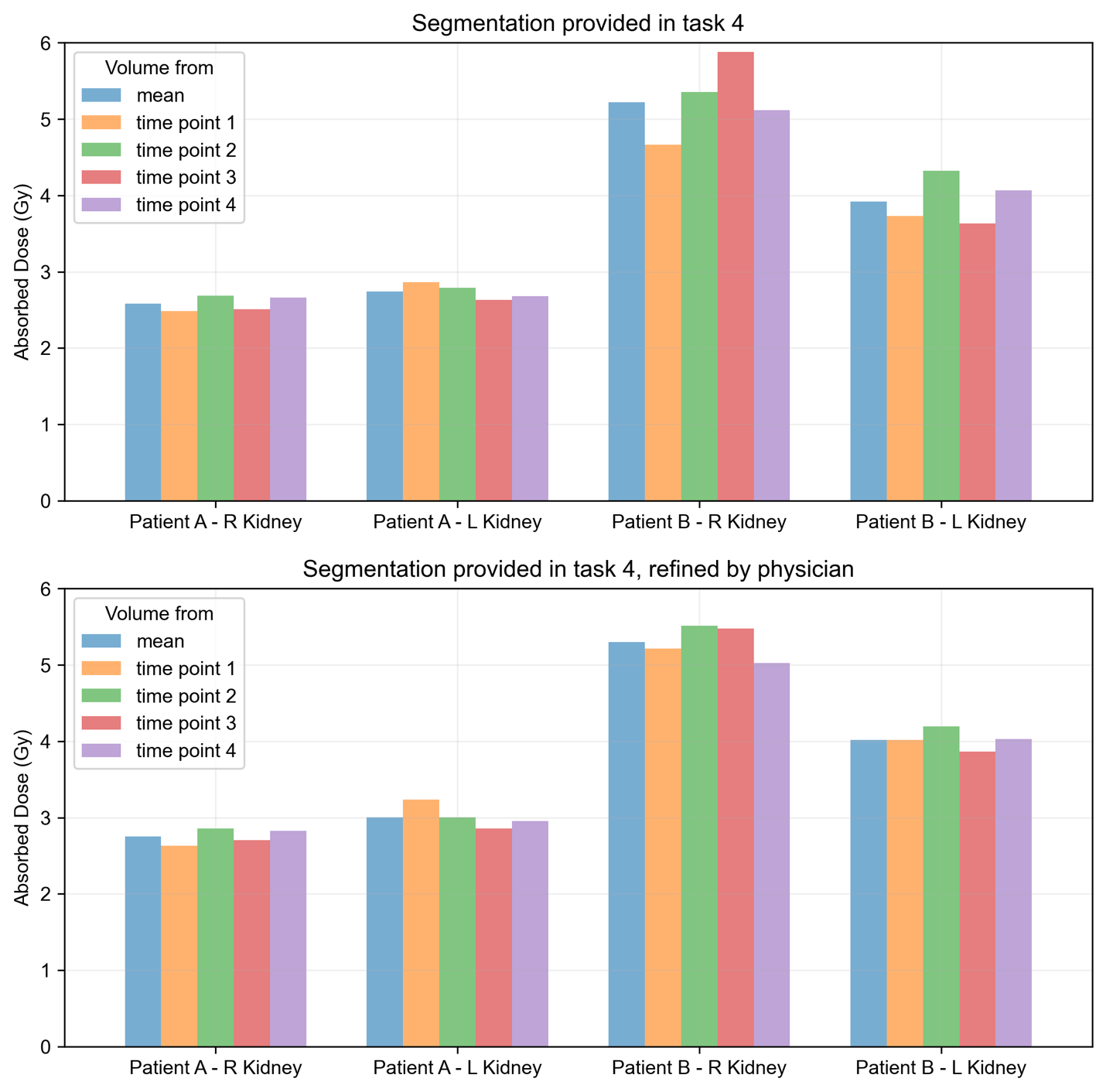


**Supplemental Figure 3**: Absorbed doses for kidneys evaluated using the segmentation provided in task4 (generated by artificial intelligence) and refined by the physician (reference method in main manuscript), with volumes derived from CT at time points 1, 2, 3, or 4, or as the mean of all volumes.

In Supplemental Figure 3, we observed greater variability in absorbed doses calculated using volumes from different time points for VOIs generated by AI compared to those additionally refined by a physician, particularly for Patient B. This discrepancy may be due to missing portions of the organ, when no refinment was performed as shown in Supplemental Figure 5.


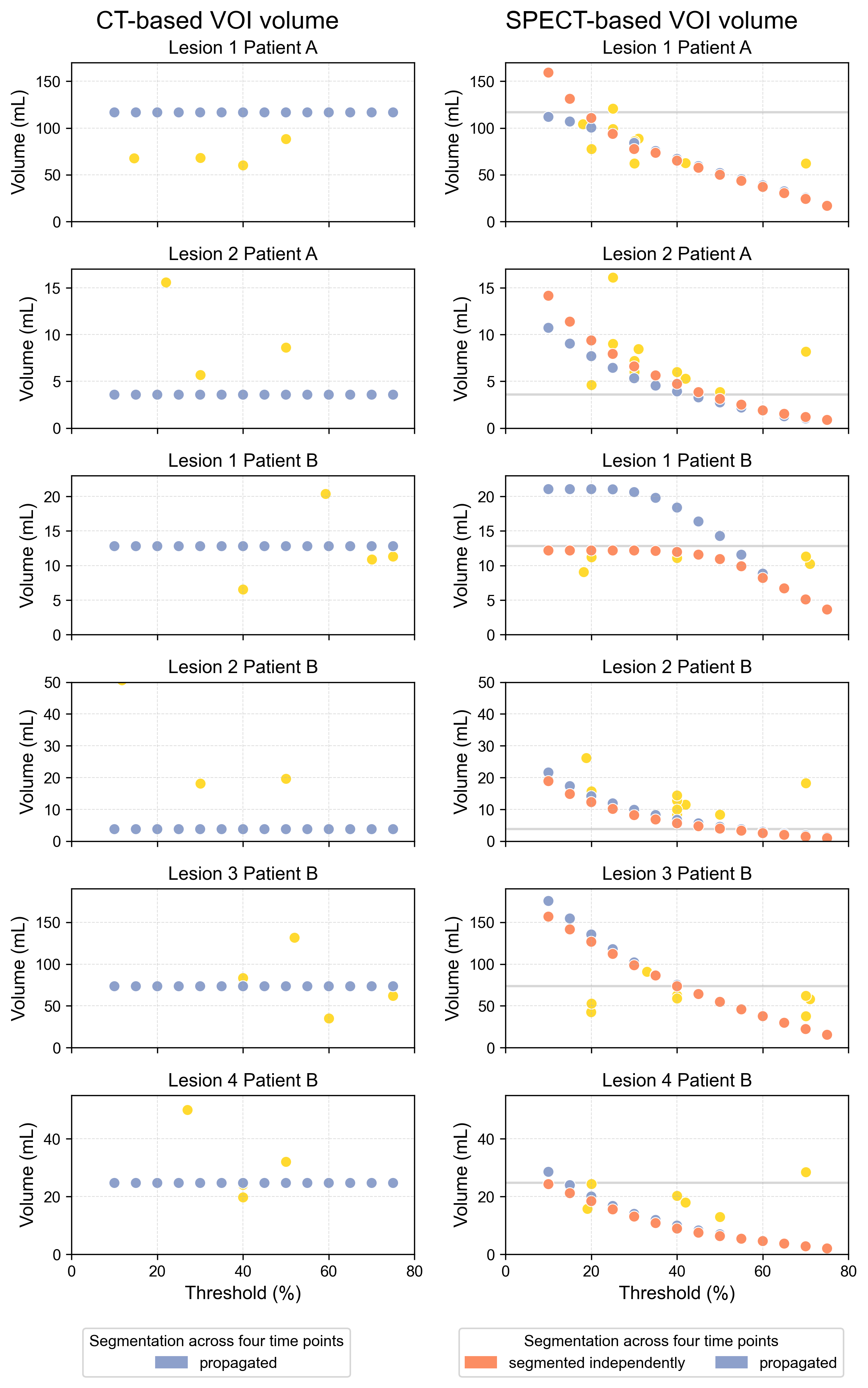


**Supplemental Figure 4**: Results of volumes generated by the authors showing the impact of different thresholding methods on volumes. The left column shows constant volume estimated using VOIs provided in task 4, while the right column shows estimation from VOIs defined by SPECT threshold boundaries. Blue dots represent the threshold applied to the second SPECT scan and propagated to subsequent time points, while orange dots represent thresholds applied independently to each SPECT scan. Yellow dots indicate participant-reported volumes from those using the threshold-based method.


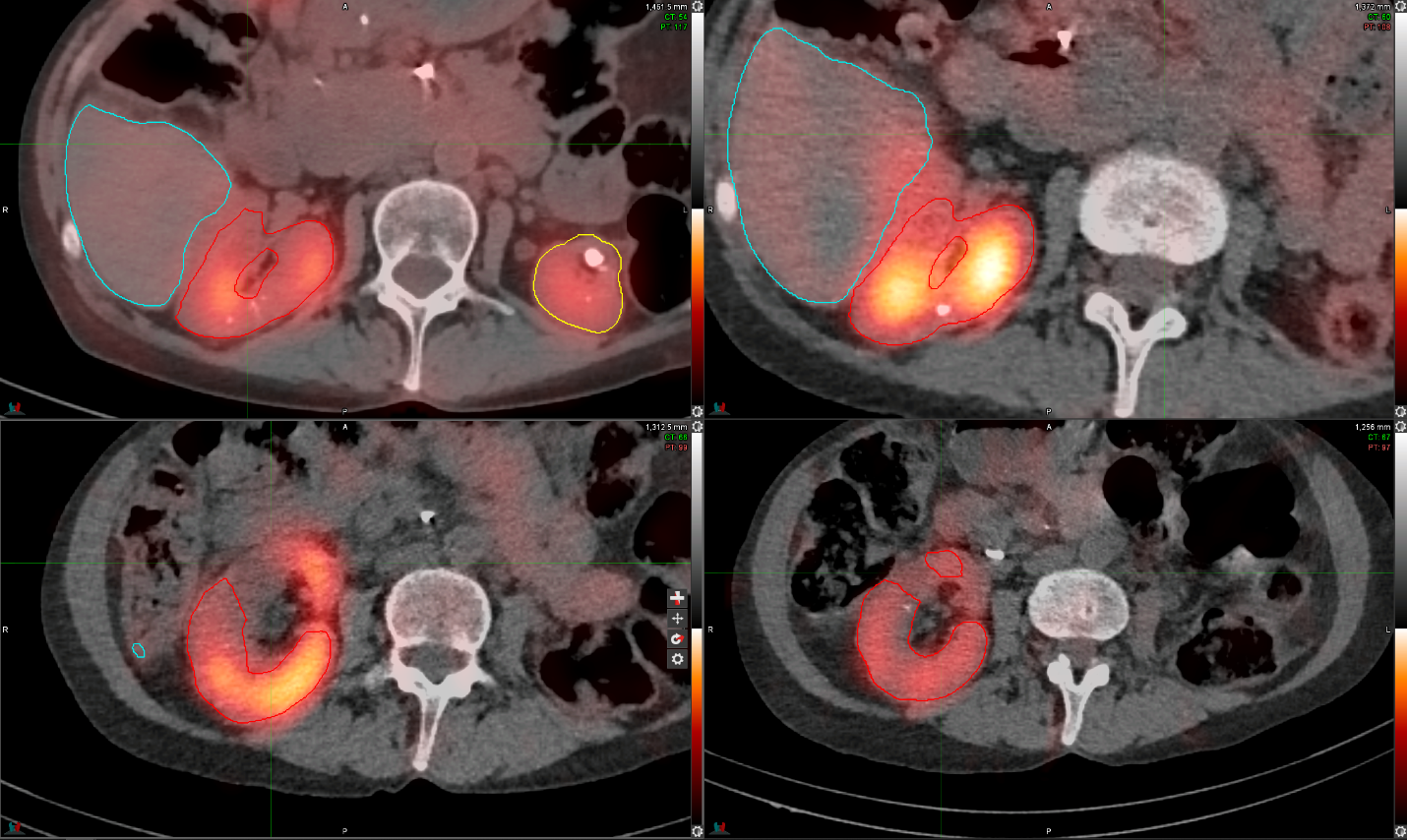

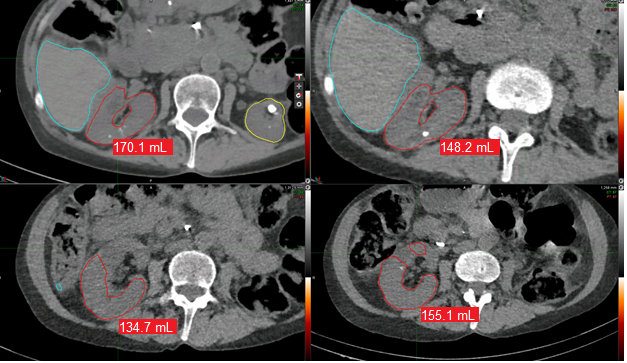


**Supplemental Figure 5**: SPECT/CT and CT images showing the right kidney of patient B, with VOIs from task 4 segmented using AI-based segmentation. Upper left (1st time point), upper right (2nd time point), lower left (3rd time point), lower right (4th time point). Parts of the kidney parenchyma are not included in the VOI near the liver (shown in the upper plots), and renal parenchyma anterior to cysts is missing (shown in the lower plots). The volumes at different time points were 170.1, 148.2, 134.7, and 155.1 mL. The later time points show volumes up to 20% smaller, primarily due to missing portions of the organ.
